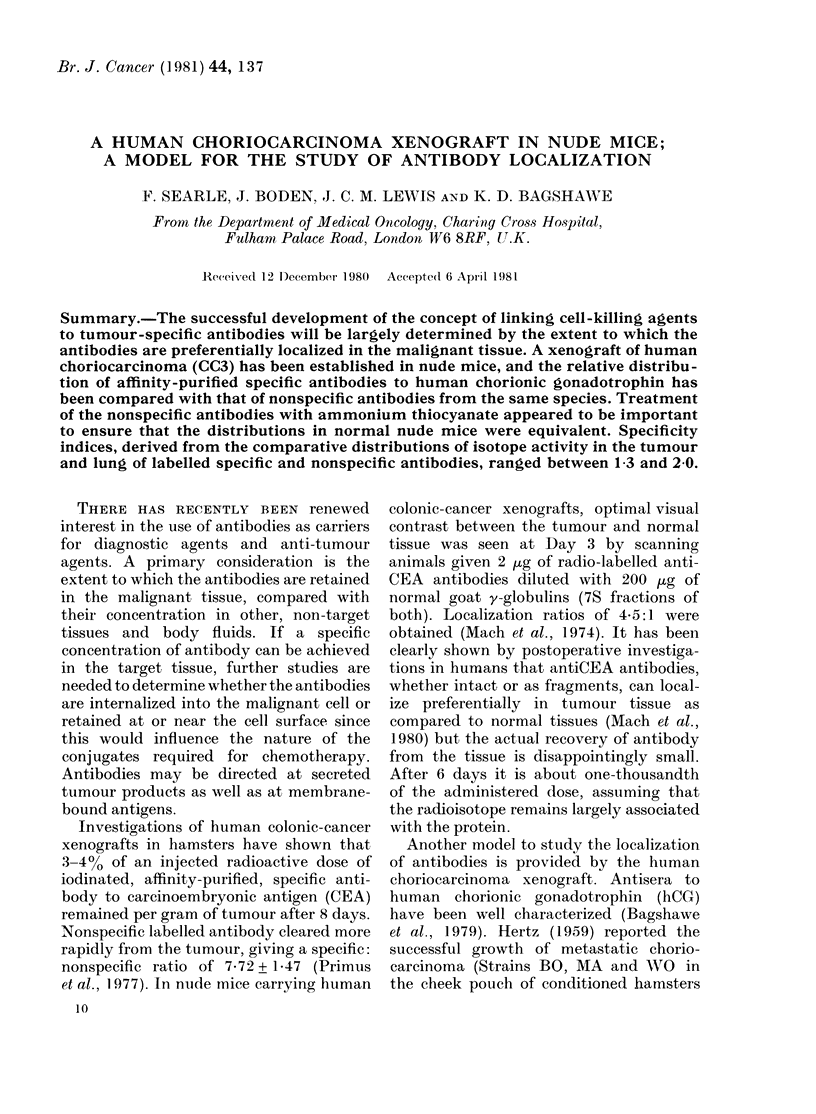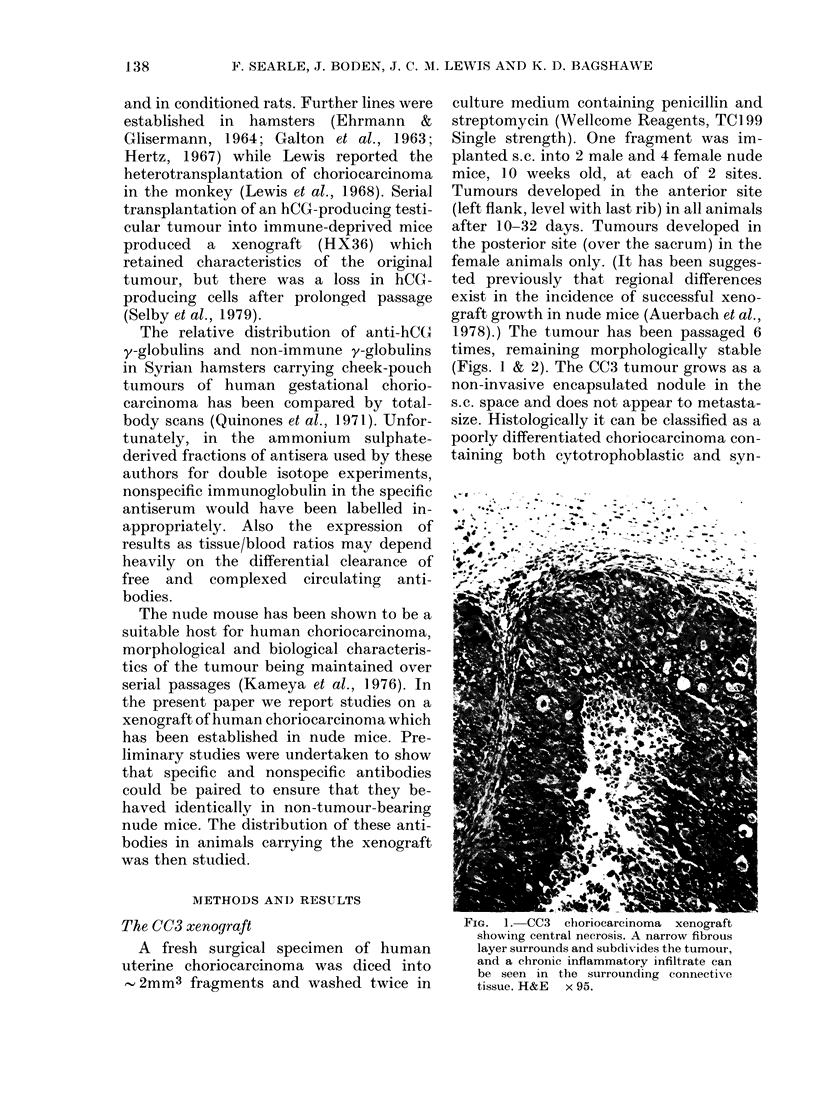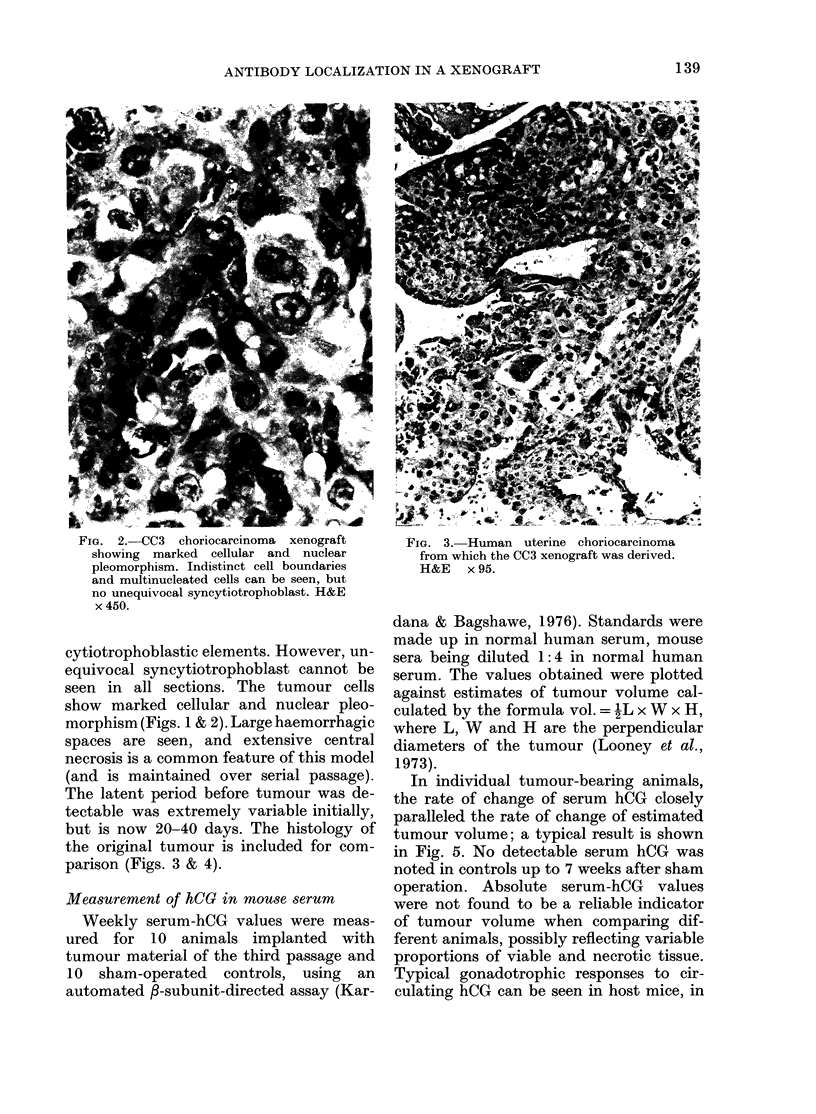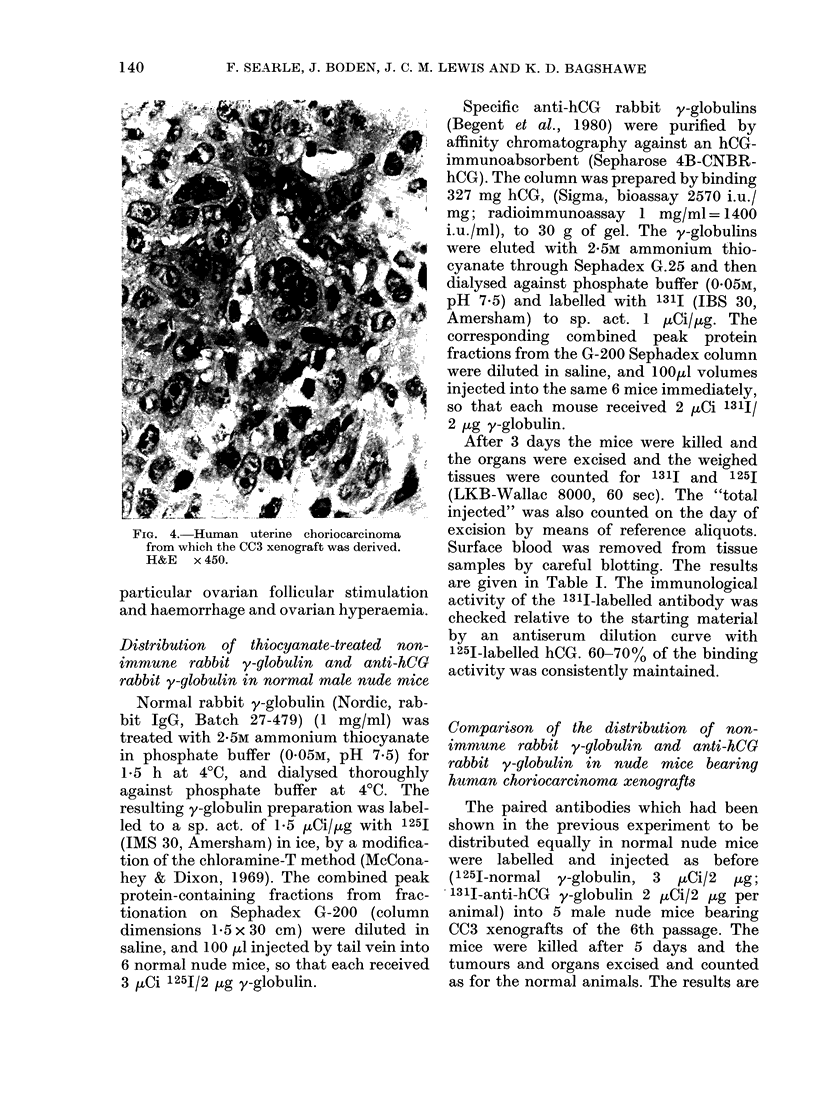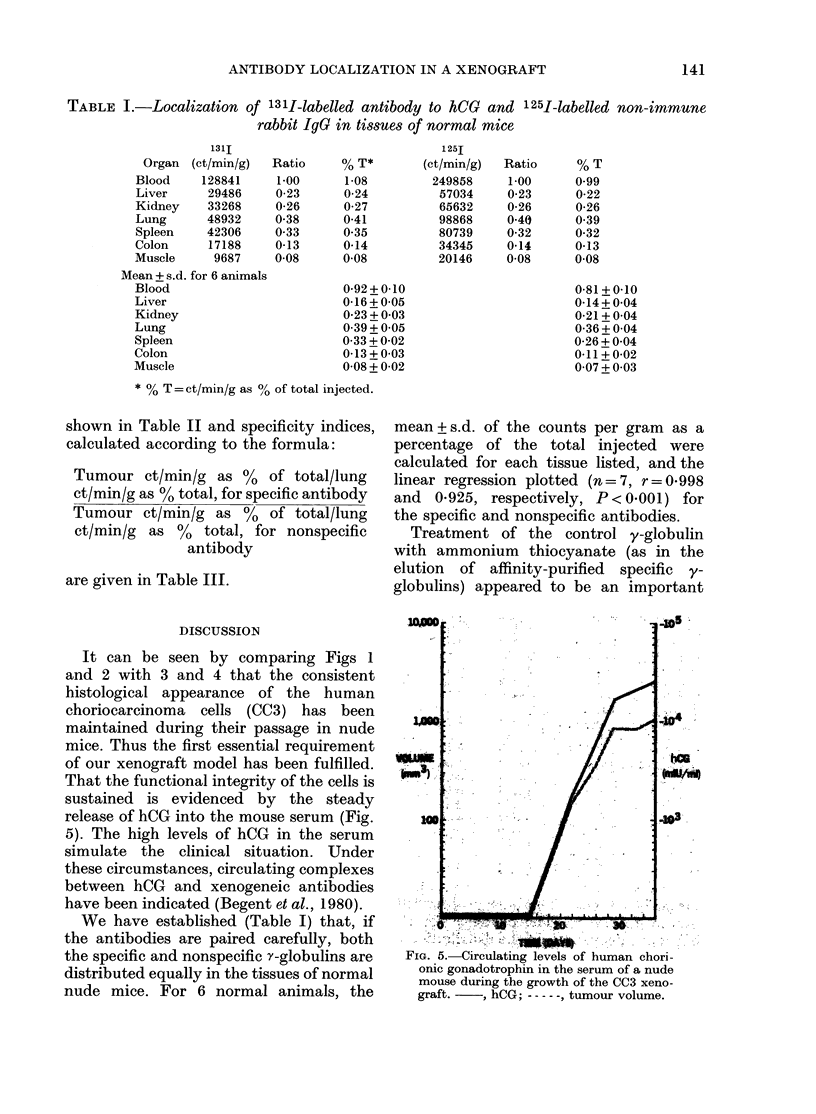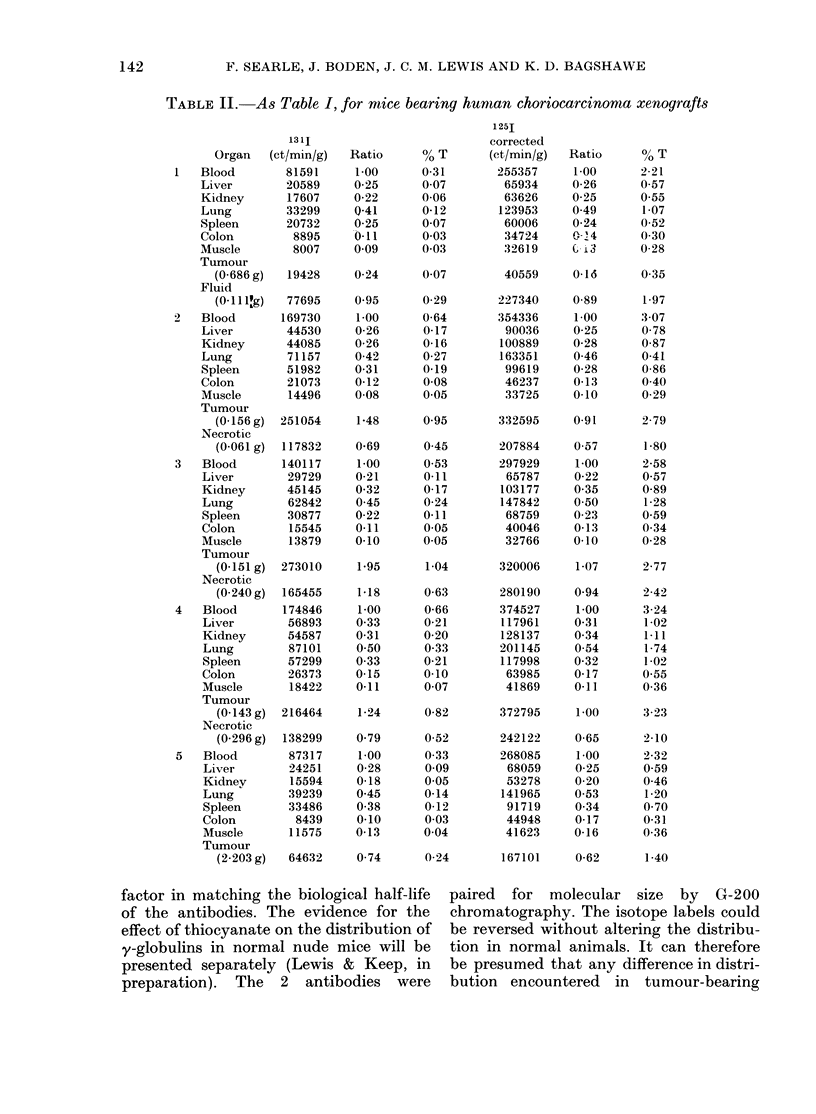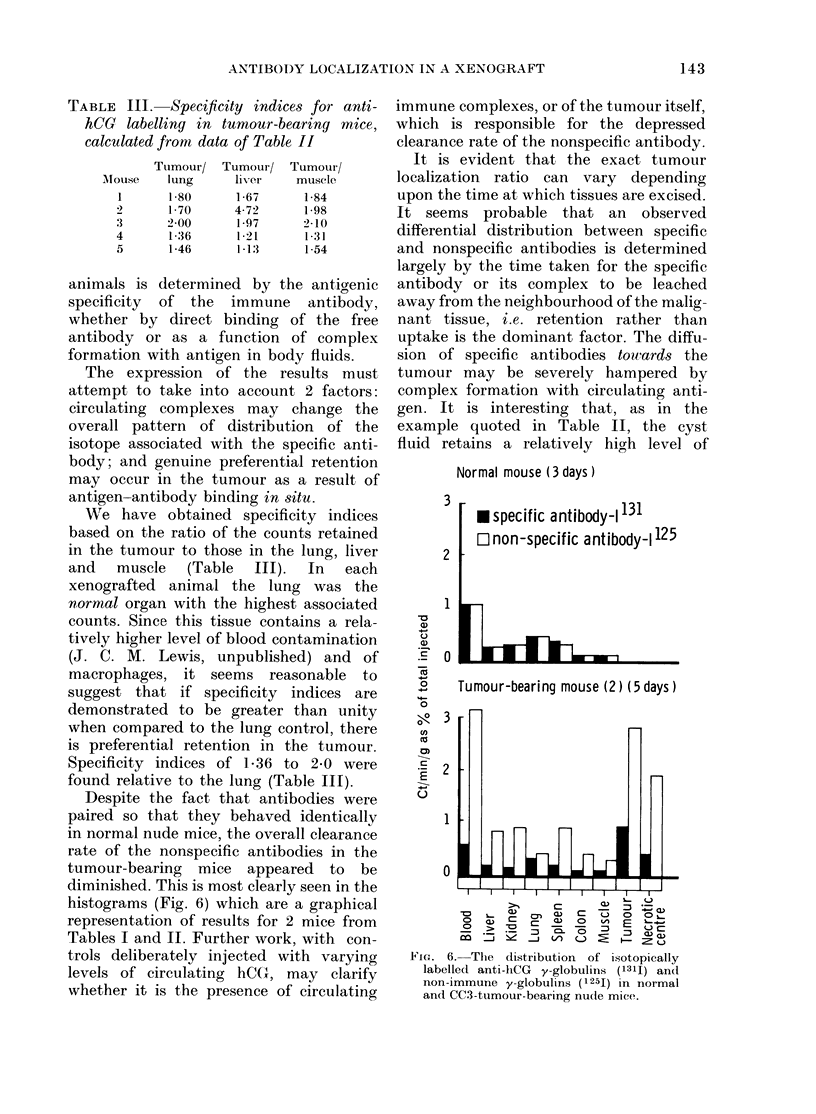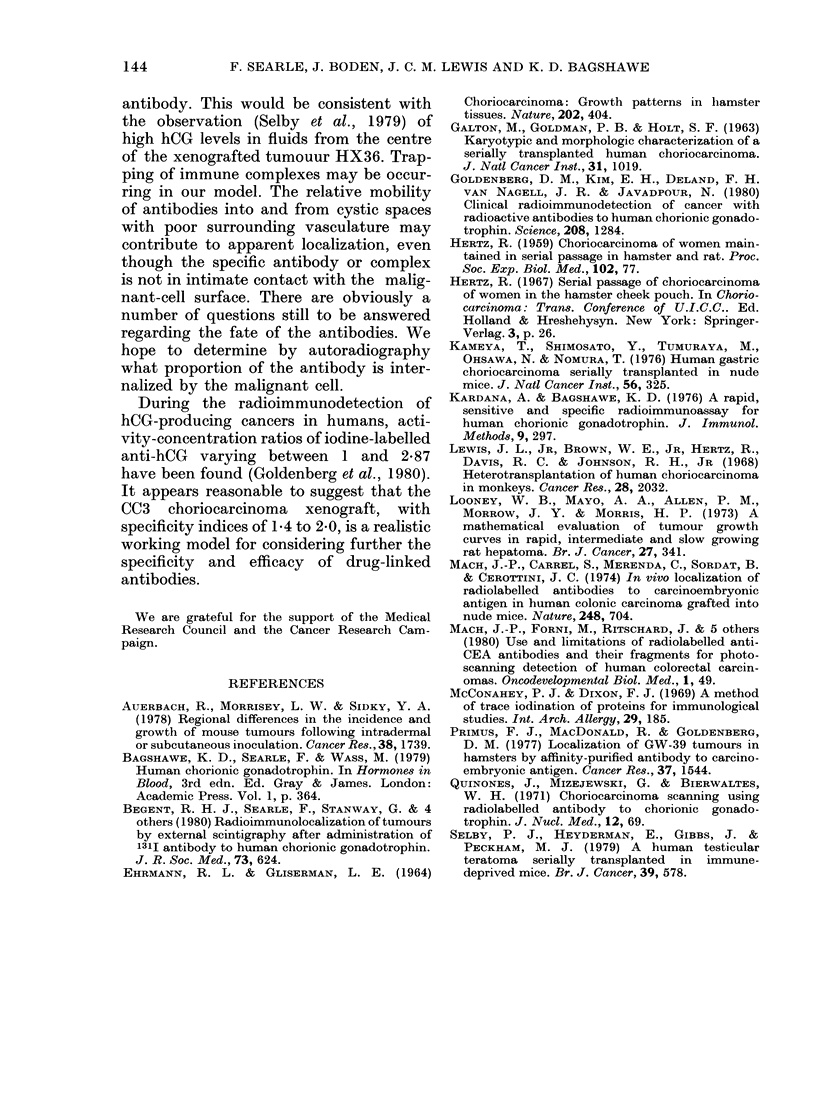# A human choriocarcinoma xenograft in nude mice; a model for the study of antibody localization.

**DOI:** 10.1038/bjc.1981.163

**Published:** 1981-08

**Authors:** F. Searle, J. Boden, J. C. Lewis, K. D. Bagshawe

## Abstract

**Images:**


*Br. J. Cancer* (1981) **44**, 137

### A HUMAN CHORIOCARCINOMA XENOGRAFT IN NUDE MICE; A MODEL FOR THE STUDY OF ANTIBODY LOCALIZATION

F. SEARLE, J. BODEN, J. C. M. LEWIS AND K. D. BAGSHAWE

*From the Department of Medical Oncology, Charing Cross Hospital, Fulham Palace Road, London W6 8RF, U.K.*

Received 12 December 1980 Accepted 6 April 1981

**Summary.—The successful development of the concept of linking cell-killing agents to tumour-specific antibodies will be largely determined by the extent to which the antibodies are preferentially localized in the malignant tissue. A xenograft of human choriocarcinoma (CC3) has been established in nude mice, and the relative distribution of affinity-purified specific antibodies to human chorionic gonadotrophin has been compared with that of nonspecific antibodies from the same species. Treatment of the nonspecific antibodies with ammonium thiocyanate appeared to be important to ensure that the distributions in normal nude mice were equivalent. Specificity indices, derived from the comparative distributions of isotope activity in the tumour and lung of labelled specific and nonspecific antibodies, ranged between 1·3 and 2·0.**

THERE HAS RECENTLY BEEN renewed interest in the use of antibodies as carriers for diagnostic agents and anti-tumour agents. A primary consideration is the extent to which the antibodies are retained in the malignant tissue, compared with their concentration in other, non-target tissues and body fluids. If a specific concentration of antibody can be achieved in the target tissue, further studies are needed to determine whether the antibodies are internalized into the malignant cell or retained at or near the cell surface since this would influence the nature of the conjugates required for chemotherapy. Antibodies may be directed at secreted tumour products as well as at membrane-bound antigens.

Investigations of human colonic-cancer xenografts in hamsters have shown that 3–4% of an injected radioactive dose of iodinated, affinity-purified, specific antibody to carcinoembryonic antigen (CEA) remained per gram of tumour after 8 days. Nonspecific labelled antibody cleared more rapidly from the tumour, giving a specific: nonspecific ratio of $7{\cdot}72 \pm 1{\cdot}47$ (Primus *et al.*, 1977). In nude mice carrying human colonic-cancer xenografts, optimal visual contrast between the tumour and normal tissue was seen at Day 3 by scanning animals given 2 $\mu$g of radio-labelled anti-CEA antibodies diluted with 200 $\mu$g of normal goat $\gamma$-globulins (7S fractions of both). Localization ratios of 4·5:1 were obtained (Mach *et al.*, 1974). It has been clearly shown by postoperative investigations in humans that antiCEA antibodies, whether intact or as fragments, can localize preferentially in tumour tissue as compared to normal tissues (Mach *et al.*, 1980) but the actual recovery of antibody from the tissue is disappointingly small. After 6 days it is about one-thousandth of the administered dose, assuming that the radioisotope remains largely associated with the protein.

Another model to study the localization of antibodies is provided by the human choriocarcinoma xenograft. Antisera to human chorionic gonadotrophin (hCG) have been well characterized (Bagshawe *et al.*, 1979). Hertz (1959) reported the successful growth of metastatic choriocarcinoma (Strains BO, MA and WO in the cheek pouch of conditioned hamsters and in conditioned rats. Further lines were established in hamsters (Ehrmann & Glisermann, 1964; Galton *et al.*, 1963; Hertz, 1967) while Lewis reported the heterotransplantation of choriocarcinoma in the monkey (Lewis *et al.*, 1968). Serial transplantation of an hCG-producing testicular tumour into immune-deprived mice produced a xenograft (HX36) which retained characteristics of the original tumour, but there was a loss in hCG-producing cells after prolonged passage (Selby *et al.*, 1979).

The relative distribution of anti-hCG $\gamma$-globulins and non-immune $\gamma$-globulins in Syrian hamsters carrying cheek-pouch tumours of human gestational choriocarcinoma has been compared by total-body scans (Quinones *et al.*, 1971). Unfortunately, in the ammonium sulphate-derived fractions of antisera used by these authors for double isotope experiments, nonspecific immunoglobulin in the specific antiserum would have been labelled inappropriately. Also the expression of results as tissue/blood ratios may depend heavily on the differential clearance of free and complexed circulating antibodies.

The nude mouse has been shown to be a suitable host for human choriocarcinoma, morphological and biological characteristics of the tumour being maintained over serial passages (Kameya *et al.*, 1976). In the present paper we report studies on a xenograft of human choriocarcinoma which has been established in nude mice. Preliminary studies were undertaken to show that specific and nonspecific antibodies could be paired to ensure that they behaved identically in non-tumour-bearing nude mice. The distribution of these antibodies in animals carrying the xenograft was then studied.

### METHODS AND RESULTS

### *The CC3 xenograft*

A fresh surgical specimen of human uterine choriocarcinoma was diced into $\sim 2mm^3$ fragments and washed twice in culture medium containing penicillin and streptomycin (Wellcome Reagents, TC199 Single strength). One fragment was implanted s.c. into 2 male and 4 female nude mice, 10 weeks old, at each of 2 sites. Tumours developed in the anterior site (left flank, level with last rib) in all animals after 10–32 days. Tumours developed in the posterior site (over the sacrum) in the female animals only. (It has been suggested previously that regional differences exist in the incidence of successful xenograft growth in nude mice (Auerbach *et al.*, 1978).) The tumour has been passaged 6 times, remaining morphologically stable (Figs. 1 & 2). The CC3 tumour grows as a non-invasive encapsulated nodule in the s.c. space and does not appear to metastasize. Histologically it can be classified as a poorly differentiated choriocarcinoma containing both cytotrophoblastic and syn-

FIG. 1.—CC3 choriocarcinoma xenograft showing central necrosis. A narrow fibrous layer surrounds and subdivides the tumour, and a chronic inflammatory infiltrate can be seen in the surrounding connective tissue. H&E ×95.

FIG. 2.—CC3 choriocarcinoma xenograft showing marked cellular and nuclear pleomorphism. Indistinct cell boundaries and multinucleated cells can be seen, but no unequivocal syncytiotrophoblast. H&E ×450.

FIG. 3.—Human uterine choriocarcinoma from which the CC3 xenograft was derived. H&E ×95.

cytiotrophoblastic elements. However, unequivocal syncytiotrophoblast cannot be seen in all sections. The tumour cells show marked cellular and nuclear pleomorphism (Figs. 1 & 2). Large haemorrhagic spaces are seen, and extensive central necrosis is a common feature of this model (and is maintained over serial passage). The latent period before tumour was detectable was extremely variable initially, but is now 20–40 days. The histology of the original tumour is included for comparison (Figs. 3 & 4).

*Measurement of hCG in mouse serum*

Weekly serum-hCG values were measured for 10 animals implanted with tumour material of the third passage and 10 sham-operated controls, using an automated $\beta$-subunit-directed assay (Kardana & Bagshawe, 1976). Standards were made up in normal human serum, mouse sera being diluted 1:4 in normal human serum. The values obtained were plotted against estimates of tumour volume calculated by the formula vol. $= \frac{1}{2}L \times W \times H$, where L, W and H are the perpendicular diameters of the tumour (Looney *et al.*, 1973).

In individual tumour-bearing animals, the rate of change of serum hCG closely paralleled the rate of change of estimated tumour volume; a typical result is shown in Fig. 5. No detectable serum hCG was noted in controls up to 7 weeks after sham operation. Absolute serum-hCG values were not found to be a reliable indicator of tumour volume when comparing different animals, possibly reflecting variable proportions of viable and necrotic tissue. Typical gonadotrophic responses to circulating hCG can be seen in host mice, in

FIG. 4.—Human uterine choriocarcinoma from which the CC3 xenograft was derived. H&E ×450.

particular ovarian follicular stimulation and haemorrhage and ovarian hyperaemia.

*Distribution of thiocyanate-treated non-immune rabbit γ-globulin and anti-hCG rabbit γ-globulin in normal male nude mice*

Normal rabbit γ-globulin (Nordic, rabbit IgG, Batch 27-479) (1 mg/ml) was treated with 2·5M ammonium thiocyanate in phosphate buffer (0·05M, pH 7·5) for 1·5 h at 4°C, and dialysed thoroughly against phosphate buffer at 4°C. The resulting γ-globulin preparation was labelled to a sp. act. of 1·5 μCi/μg with $^{125}$I (IMS 30, Amersham) in ice, by a modification of the chloramine-T method (McConahey & Dixon, 1969). The combined peak protein-containing fractions from fractionation on Sephadex G-200 (column dimensions 1·5 × 30 cm) were diluted in saline, and 100 μl injected by tail vein into 6 normal nude mice, so that each received 3 μCi $^{125}$I/2 μg γ-globulin.

Specific anti-hCG rabbit γ-globulins (Begent *et al.*, 1980) were purified by affinity chromatography against an hCG-immunoabsorbent (Sepharose 4B-CNBR-hCG). The column was prepared by binding 327 mg hCG, (Sigma, bioassay 2570 i.u./mg; radioimmunoassay 1 mg/ml = 1400 i.u./ml), to 30 g of gel. The γ-globulins were eluted with 2·5M ammonium thiocyanate through Sephadex G.25 and then dialysed against phosphate buffer (0·05M, pH 7·5) and labelled with $^{131}$I (IBS 30, Amersham) to sp. act. 1 μCi/μg. The corresponding combined peak protein fractions from the G-200 Sephadex column were diluted in saline, and 100μl volumes injected into the same 6 mice immediately, so that each mouse received 2 μCi $^{131}$I/2 μg γ-globulin.

After 3 days the mice were killed and the organs were excised and the weighed tissues were counted for $^{131}$I and $^{125}$I (LKB-Wallac 8000, 60 sec). The "total injected" was also counted on the day of excision by means of reference aliquots. Surface blood was removed from tissue samples by careful blotting. The results are given in Table I. The immunological activity of the $^{131}$I-labelled antibody was checked relative to the starting material by an antiserum dilution curve with $^{125}$I-labelled hCG. 60–70% of the binding activity was consistently maintained.

*Comparison of the distribution of non-immune rabbit γ-globulin and anti-hCG rabbit γ-globulin in nude mice bearing human choriocarcinoma xenografts*

The paired antibodies which had been shown in the previous experiment to be distributed equally in normal nude mice were labelled and injected as before ($^{125}$I-normal γ-globulin, 3 μCi/2 μg; $^{131}$I-anti-hCG γ-globulin 2 μCi/2 μg per animal) into 5 male nude mice bearing CC3 xenografts of the 6th passage. The mice were killed after 5 days and the tumours and organs excised and counted as for the normal animals. The results are

TABLE I.—*Localization of $^{131}$I-labelled antibody to hCG and $^{125}$I-labelled non-immune rabbit IgG in tissues of normal mice*

| Organ | $^{131}$I (ct/min/g) | Ratio | % T* | $^{125}$I (ct/min/g) | Ratio | % T |
|---|---|---|---|---|---|---|
| Blood | 128841 | 1·00 | 1·08 | 249858 | 1·00 | 0·99 |
| Liver | 29486 | 0·23 | 0·24 | 57034 | 0·23 | 0·22 |
| Kidney | 33268 | 0·26 | 0·27 | 65632 | 0·26 | 0·26 |
| Lung | 48932 | 0·38 | 0·41 | 98868 | 0·40 | 0·39 |
| Spleen | 42306 | 0·33 | 0·35 | 80739 | 0·32 | 0·32 |
| Colon | 17188 | 0·13 | 0·14 | 34345 | 0·14 | 0·13 |
| Muscle | 9687 | 0·08 | 0·08 | 20146 | 0·08 | 0·08 |
| Mean ± s.d. for 6 animals | | | | | | |
| Blood | | | 0·92 ± 0·10 | | | 0·81 ± 0·10 |
| Liver | | | 0·16 ± 0·05 | | | 0·14 ± 0·04 |
| Kidney | | | 0·23 ± 0·03 | | | 0·21 ± 0·04 |
| Lung | | | 0·39 ± 0·05 | | | 0·36 ± 0·04 |
| Spleen | | | 0·33 ± 0·02 | | | 0·26 ± 0·04 |
| Colon | | | 0·13 ± 0·03 | | | 0·11 ± 0·02 |
| Muscle | | | 0·08 ± 0·02 | | | 0·07 ± 0·03 |

* % T = ct/min/g as % of total injected.

shown in Table II and specificity indices, calculated according to the formula:

$$\frac{\text{Tumour ct/min/g as \% of total/lung ct/min/g as \% total, for specific antibody}}{\text{Tumour ct/min/g as \% of total/lung ct/min/g as \% total, for nonspecific antibody}}$$

are given in Table III.

DISCUSSION

It can be seen by comparing Figs 1 and 2 with 3 and 4 that the consistent histological appearance of the human choriocarcinoma cells (CC3) has been maintained during their passage in nude mice. Thus the first essential requirement of our xenograft model has been fulfilled. That the functional integrity of the cells is sustained is evidenced by the steady release of hCG into the mouse serum (Fig. 5). The high levels of hCG in the serum simulate the clinical situation. Under these circumstances, circulating complexes between hCG and xenogeneic antibodies have been indicated (Begent *et al.*, 1980).

We have established (Table I) that, if the antibodies are paired carefully, both the specific and nonspecific γ-globulins are distributed equally in the tissues of normal nude mice. For 6 normal animals, the mean ± s.d. of the counts per gram as a percentage of the total injected were calculated for each tissue listed, and the linear regression plotted ($n = 7$, $r = 0{\cdot}998$ and $0{\cdot}925$, respectively, $P < 0{\cdot}001$) for the specific and nonspecific antibodies.

Treatment of the control γ-globulin with ammonium thiocyanate (as in the elution of affinity-purified specific γ-globulins) appeared to be an important

FIG. 5.—Circulating levels of human chorionic gonadotrophin in the serum of a nude mouse during the growth of the CC3 xenograft. ——, hCG; - - - - -, tumour volume.

TABLE II.—*As Table I, for mice bearing human choriocarcinoma xenografts*

| | Organ | $^{131}$I (ct/min/g) | Ratio | % T | $^{125}$I corrected (ct/min/g) | Ratio | % T |
|---|---|---|---|---|---|---|---|
| 1 | Blood | 81591 | 1·00 | 0·31 | 255357 | 1·00 | 2·21 |
| | Liver | 20589 | 0·25 | 0·07 | 65934 | 0·26 | 0·57 |
| | Kidney | 17607 | 0·22 | 0·06 | 63626 | 0·25 | 0·55 |
| | Lung | 33299 | 0·41 | 0·12 | 123953 | 0·49 | 1·07 |
| | Spleen | 20732 | 0·25 | 0·07 | 60006 | 0·24 | 0·52 |
| | Colon | 8895 | 0·11 | 0·03 | 34724 | 0·14 | 0·30 |
| | Muscle | 8007 | 0·09 | 0·03 | 32619 | 0·13 | 0·28 |
| | Tumour (0·686 g) | 19428 | 0·24 | 0·07 | 40559 | 0·16 | 0·35 |
| | Fluid (0·111 g) | 77695 | 0·95 | 0·29 | 227340 | 0·89 | 1·97 |
| 2 | Blood | 169730 | 1·00 | 0·64 | 354336 | 1·00 | 3·07 |
| | Liver | 44530 | 0·26 | 0·17 | 90036 | 0·25 | 0·78 |
| | Kidney | 44085 | 0·26 | 0·16 | 100889 | 0·28 | 0·87 |
| | Lung | 71157 | 0·42 | 0·27 | 163351 | 0·46 | 0·41 |
| | Spleen | 51982 | 0·31 | 0·19 | 99619 | 0·28 | 0·86 |
| | Colon | 21073 | 0·12 | 0·08 | 46237 | 0·13 | 0·40 |
| | Muscle | 14496 | 0·08 | 0·05 | 33725 | 0·10 | 0·29 |
| | Tumour (0·156 g) | 251054 | 1·48 | 0·95 | 332595 | 0·91 | 2·79 |
| | Necrotic (0·061 g) | 117832 | 0·69 | 0·45 | 207884 | 0·57 | 1·80 |
| 3 | Blood | 140117 | 1·00 | 0·53 | 297929 | 1·00 | 2·58 |
| | Liver | 29729 | 0·21 | 0·11 | 65787 | 0·22 | 0·57 |
| | Kidney | 45145 | 0·32 | 0·17 | 103177 | 0·35 | 0·89 |
| | Lung | 62842 | 0·45 | 0·24 | 147842 | 0·50 | 1·28 |
| | Spleen | 30877 | 0·22 | 0·11 | 68759 | 0·23 | 0·59 |
| | Colon | 15545 | 0·11 | 0·05 | 40046 | 0·13 | 0·34 |
| | Muscle | 13879 | 0·10 | 0·05 | 32766 | 0·10 | 0·28 |
| | Tumour (0·151 g) | 273010 | 1·95 | 1·04 | 320006 | 1·07 | 2·77 |
| | Necrotic (0·240 g) | 165455 | 1·18 | 0·63 | 280190 | 0·94 | 2·42 |
| 4 | Blood | 174846 | 1·00 | 0·66 | 374527 | 1·00 | 3·24 |
| | Liver | 56893 | 0·33 | 0·21 | 117961 | 0·31 | 1·02 |
| | Kidney | 54587 | 0·31 | 0·20 | 128137 | 0·34 | 1·11 |
| | Lung | 87101 | 0·50 | 0·33 | 201145 | 0·54 | 1·74 |
| | Spleen | 57299 | 0·33 | 0·21 | 117998 | 0·32 | 1·02 |
| | Colon | 26373 | 0·15 | 0·10 | 63985 | 0·17 | 0·55 |
| | Muscle | 18422 | 0·11 | 0·07 | 41869 | 0·11 | 0·36 |
| | Tumour (0·143 g) | 216464 | 1·24 | 0·82 | 372795 | 1·00 | 3·23 |
| | Necrotic (0·296 g) | 138299 | 0·79 | 0·52 | 242122 | 0·65 | 2·10 |
| 5 | Blood | 87317 | 1·00 | 0·33 | 268085 | 1·00 | 2·32 |
| | Liver | 24251 | 0·28 | 0·09 | 68059 | 0·25 | 0·59 |
| | Kidney | 15594 | 0·18 | 0·05 | 53278 | 0·20 | 0·46 |
| | Lung | 39239 | 0·45 | 0·14 | 141965 | 0·53 | 1·20 |
| | Spleen | 33486 | 0·38 | 0·12 | 91719 | 0·34 | 0·70 |
| | Colon | 8439 | 0·10 | 0·03 | 44948 | 0·17 | 0·31 |
| | Muscle | 11575 | 0·13 | 0·04 | 41623 | 0·16 | 0·36 |
| | Tumour (2·203 g) | 64632 | 0·74 | 0·24 | 167101 | 0·62 | 1·40 |

factor in matching the biological half-life of the antibodies. The evidence for the effect of thiocyanate on the distribution of γ-globulins in normal nude mice will be presented separately (Lewis & Keep, in preparation). The 2 antibodies were paired for molecular size by G-200 chromatography. The isotope labels could be reversed without altering the distribution in normal animals. It can therefore be presumed that any difference in distribution encountered in tumour-bearing

TABLE III.—*Specificity indices for anti-hCG labelling in tumour-bearing mice, calculated from data of Table II*

| Mouse | Tumour/ lung | Tumour/ liver | Tumour/ muscle |
|---|---|---|---|
| 1 | 1·80 | 1·67 | 1·84 |
| 2 | 1·70 | 4·72 | 1·98 |
| 3 | 2·00 | 1·97 | 2·10 |
| 4 | 1·36 | 1·21 | 1·31 |
| 5 | 1·46 | 1·13 | 1·54 |

animals is determined by the antigenic specificity of the immune antibody, whether by direct binding of the free antibody or as a function of complex formation with antigen in body fluids.

The expression of the results must attempt to take into account 2 factors: circulating complexes may change the overall pattern of distribution of the isotope associated with the specific antibody; and genuine preferential retention may occur in the tumour as a result of antigen–antibody binding *in situ*.

We have obtained specificity indices based on the ratio of the counts retained in the tumour to those in the lung, liver and muscle (Table III). In each xenografted animal the lung was the *normal* organ with the highest associated counts. Since this tissue contains a relatively higher level of blood contamination (J. C. M. Lewis, unpublished) and of macrophages, it seems reasonable to suggest that if specificity indices are demonstrated to be greater than unity when compared to the lung control, there is preferential retention in the tumour. Specificity indices of 1·36 to 2·0 were found relative to the lung (Table III).

Despite the fact that antibodies were paired so that they behaved identically in normal nude mice, the overall clearance rate of the nonspecific antibodies in the tumour-bearing mice appeared to be diminished. This is most clearly seen in the histograms (Fig. 6) which are a graphical representation of results for 2 mice from Tables I and II. Further work, with controls deliberately injected with varying levels of circulating hCG, may clarify whether it is the presence of circulating immune complexes, or of the tumour itself, which is responsible for the depressed clearance rate of the nonspecific antibody.

It is evident that the exact tumour localization ratio can vary depending upon the time at which tissues are excised. It seems probable that an observed differential distribution between specific and nonspecific antibodies is determined largely by the time taken for the specific antibody or its complex to be leached away from the neighbourhood of the malignant tissue, *i.e.* retention rather than uptake is the dominant factor. The diffusion of specific antibodies *towards* the tumour may be severely hampered by complex formation with circulating antigen. It is interesting that, as in the example quoted in Table II, the cyst fluid retains a relatively high level of

FIG. 6.—The distribution of isotopically labelled anti-hCG γ-globulins ($^{131}I$) and non-immune γ-globulins ($^{125}I$) in normal and CC3-tumour-bearing nude mice.

antibody. This would be consistent with the observation (Selby *et al.*, 1979) of high hCG levels in fluids from the centre of the xenografted tumouur HX36. Trapping of immune complexes may be occurring in our model. The relative mobility of antibodies into and from cystic spaces with poor surrounding vasculature may contribute to apparent localization, even though the specific antibody or complex is not in intimate contact with the malignant-cell surface. There are obviously a number of questions still to be answered regarding the fate of the antibodies. We hope to determine by autoradiography what proportion of the antibody is internalized by the malignant cell.

During the radioimmunodetection of hCG-producing cancers in humans, activity-concentration ratios of iodine-labelled anti-hCG varying between 1 and 2·87 have been found (Goldenberg *et al.*, 1980). It appears reasonable to suggest that the CC3 choriocarcinoma xenograft, with specificity indices of 1·4 to 2·0, is a realistic working model for considering further the specificity and efficacy of drug-linked antibodies.

We are grateful for the support of the Medical Research Council and the Cancer Research Campaign.

### REFERENCES

AUERBACH, R., MORRISEY, L. W. & SIDKY, Y. A. (1978) Regional differences in the incidence and growth of mouse tumours following intradermal or subcutaneous inoculation. *Cancer Res.*, **38**, 1739.

BAGSHAWE, K. D., SEARLE, F. & WASS, M. (1979) Human chorionic gonadotrophin. In *Hormones in Blood*, 3rd edn. Ed. Gray & James. London: Academic Press. Vol. 1, p. 364.

BEGENT, R. H. J., SEARLE, F., STANWAY, G. & 4 others (1980) Radioimmunolocalization of tumours by external scintigraphy after administration of $^{131}$I antibody to human chorionic gonadotrophin. *J. R. Soc. Med.*, **73**, 624.

EHRMANN, R. L. & GLISERMAN, L. E. (1964) Choriocarcinoma: Growth patterns in hamster tissues. *Nature*, **202**, 404.

GALTON, M., GOLDMAN, P. B. & HOLT, S. F. (1963) Karyotypic and morphologic characterization of a serially transplanted human choriocarcinoma. *J. Natl Cancer Inst.*, **31**, 1019.

GOLDENBERG, D. M., KIM, E. H., DELAND, F. H. VAN NAGELL, J. R. & JAVADPOUR, N. (1980) Clinical radioimmunodetection of cancer with radioactive antibodies to human chorionic gonadotrophin. *Science*, **208**, 1284.

HERTZ, R. (1959) Choriocarcinoma of women maintained in serial passage in hamster and rat. *Proc. Soc. Exp. Biol. Med.*, **102**, 77.

HERTZ, R. (1967) Serial passage of choriocarcinoma of women in the hamster cheek pouch. In *Choriocarcinoma: Trans. Conference of U.I.C.C.*. Ed. Holland & Hreshehysyn. New York: Springer-Verlag. **3**, p. 26.

KAMEYA, T., SHIMOSATO, Y., TUMURAYA, M., OHSAWA, N. & NOMURA, T. (1976) Human gastric choriocarcinoma serially transplanted in nude mice. *J. Natl Cancer Inst.*, **56**, 325.

KARDANA, A. & BAGSHAWE, K. D. (1976) A rapid, sensitive and specific radioimmunoassay for human chorionic gonadotrophin. *J. Immunol. Methods*, **9**, 297.

LEWIS, J. L., JR, BROWN, W. E., JR, HERTZ, R., DAVIS, R. C. & JOHNSON, R. H., JR (1968) Heterotransplantation of human choriocarcinoma in monkeys. *Cancer Res.*, **28**, 2032.

LOONEY, W. B., MAYO, A. A., ALLEN, P. M., MORROW, J. Y. & MORRIS, H. P. (1973) A mathematical evaluation of tumour growth curves in rapid, intermediate and slow growing rat hepatoma. *Br. J. Cancer*, **27**, 341.

MACH, J.-P., CARREL, S., MERENDA, C., SORDAT, B. & CEROTTINI, J. C. (1974) *In vivo* localization of radiolabelled antibodies to carcinoembryonic antigen in human colonic carcinoma grafted into nude mice. *Nature*, **248**, 704.

MACH, J.-P., FORNI, M., RITSCHARD, J. & 5 others (1980) Use and limitations of radiolabelled anti-CEA antibodies and their fragments for photoscanning detection of human colorectal carcinomas. *Oncodevelopmental Biol. Med.*, **1**, 49.

MCCONAHEY, P. J. & DIXON, F. J. (1969) A method of trace iodination of proteins for immunological studies. *Int. Arch. Allergy*, **29**, 185.

PRIMUS, F. J., MACDONALD, R. & GOLDENBERG, D. M. (1977) Localization of GW-39 tumours in hamsters by affinity-purified antibody to carcinoembryonic antigen. *Cancer Res.*, **37**, 1544.

QUINONES, J., MIZEJEWSKI, G. & BIERWALTES, W. H. (1971) Choriocarcinoma scanning using radiolabelled antibody to chorionic gonadotrophin. *J. Nucl. Med.*, **12**, 69.

SELBY, P. J., HEYDERMAN, E., GIBBS, J. & PECKHAM, M. J. (1979) A human testicular teratoma serially transplanted in immune-deprived mice. *Br. J. Cancer*, **39**, 578.